# An RNA Polymerase III General Transcription Factor Engages in Cell Type-Specific Chromatin Looping

**DOI:** 10.3390/ijms23042260

**Published:** 2022-02-18

**Authors:** Lara de Llobet Cucalon, Chiara Di Vona, Marco Morselli, Marco Vezzoli, Barbara Montanini, Martin Teichmann, Susana de la Luna, Roberto Ferrari

**Affiliations:** 1 Center for Genomic Regulation (CRG), The Barcelona Institute of Science and Technology (BIST), Dr. Aiguader 88, 08003 Barcelona, Spain; lara.llobet@crg.eu (L.d.L.C.); chiara.divona@crg.eu (C.D.V.); susana.luna@crg.eu (S.d.l.L.); 2 Centro de Investigación Biomédica en Red en Enfermedades Raras (CIBERER), 28029 Madrid, Spain; 3 Department of Chemistry, Life Sciences and Environmental, University of Parma, Parco Area delle Scienze 11/A, 43124 Parma, Italy; marco.morselli@unipr.it (M.M.); marco.vezzoli@unipr.it (M.V.); barbara.montanini@unipr.it (B.M.); 4 Bordeaux Institute of Oncology (BRIC), University of Bordeaux Inserm U1312, 33076 Bordeaux, France; martin.teichmann@inserm.fr; 5 Institució Catalana de Recerca i Estudis Avançats (ICREA), Passeig Lluís Companys 23, 08010 Barcelona, Spain

**Keywords:** TFIIIC, 3D genome, repetitive elements

## Abstract

Transcription factors (TFs) bind DNA in a sequence-specific manner and are generally cell type-specific factors and/or developmental master regulators. In contrast, general TFs (GTFs) are part of very large protein complexes and serve for RNA polymerases’ recruitment to promoter sequences, generally in a cell type-independent manner. Whereas, several TFs have been proven to serve as anchors for the 3D genome organization, the role of GTFs in genome architecture have not been carefully explored. Here, we used ChIP-seq and Hi-C data to depict the role of TFIIIC, one of the RNA polymerase III GTFs, in 3D genome organization. We find that TFIIIC genome occupancy mainly occurs at specific regions, which largely correspond to Alu elements; other characteristic classes of repetitive elements (REs) such as MIR, FLAM-C and ALR/alpha are also found depending on the cell’s developmental origin. The analysis also shows that TFIIIC-enriched regions are involved in cell type-specific DNA looping, which does not depend on colocalization with the master architectural protein CTCF. This work extends previous knowledge on the role of TFIIIC as a bona fide genome organizer whose action participates in cell type-dependent 3D genome looping via binding to REs.

## 1. Introduction

Three-dimensional folding of the mammalian genome acts as a decisive regulatory layer for determining important physiological processes such as cell fate, development, differentiation, and ultimately even pathological conditions such as tumorigenesis, by implementing cell-type specific gene expression programs [1]. Several lines of evidence point to the existence of intricate topological chromatin assemblies, in which multiple genes and their regulatory elements interact with each other in a 3D fashion. Thus, cell-type-unique gene expression programs are therefore governed by the 3D genome structure organization in mammals [2], which in turn hangs on the action of transcription factors (TFs) capable of recognizing specific DNA sequences within the genome [3]. TFs can be divided into three major categories: transcriptional activators/repressors, co-activators/co-repressors and general transcription factors (GTFs), which direct RNA polymerases (Pol) recruitment or the level of transcription (activators and coactivators). The first two classes of TFs can be expressed ubiquitously or be cell type-specific factors (CTFs), whereas GTFs are expressed in every single cell of the organism [2,3,4]. The actions of CTFs are largely studied and depend on the overwhelming number of distinct and specific DNA binding sites on regulatory regions, either in the promoter regions or tens to hundreds of kilobases away from the promoters they activate [5,6,7]. The latter are known as enhancers and/or super-enhancers and they are referred to as regulatory distal enhancers elements (DEEs). The GTFs recognize specific regulatory sequences, so called core promoter elements (CPEs), in close proximity of the transcription start site (TTS) [8], which are capable of directly triggering the recruitment of Pols to the TSS of genes.

DEEs are loaded with a plethora of CTFs [9], which ultimately impose certain promoters to be selectively silenced or activated [10]. DEEs and their target promoters could potentially interact via the action of CTFs within larger chromosomal loop structures, formed by the interaction of architectural proteins (such as CCCTC-binding factor (CTCF), Cohesin or Condensin) bound to each of the loop anchors [11]. Indeed, recent work has brought to light how CTFs participate in cell fate determination by virtue of their ability to anchor regulatory elements such as DEEs and gene promoters [12]. In this view, sequence-specific CTFs occupying DEEs become new players in shaping the 3D genome organization and consequently, in the acquisition of transcription programs that define cell identity. However, despite the foundational importance of proper gene control to rule cell identity and organism development, a more detailed view on how CTFs contribute to structural interactions between DEEs and promoters remains poorly characterized.

A generally accepted model for the communication between DEEs and CPEs proposes that CTFs and GTFs are linked by a bridge such as the Mediator coactivator complex [13], pointing to GTFs as potential new players in genome 3D organization. This is particularly remarkable based on findings by which the basic dichotomy of cis-regulatory elements has been challenged by broad similarities between genetic and epigenetic properties of promoters (and their CPEs) and DEEs, including recruitment of Pol II machinery and its GTFs, divergent transcription initiation, production of short enhancer RNAs (eRNAs) and context dependent histone modifications [14,15]. Moreover, genomic and proteomic experiments have shed light on the unusual genomic occupancy of some GTFs to DEEs and correlated their occupancy with typical epigenetic profiling of enhancer elements [16,17]. Not surprisingly, some of the GTFs identified as novel DEE regulators also harbor their own histone modifying activity [18,19]. In this context, one of our recent works has unveiled a crucial role of a Pol III GTF, namely transcription factor C (TFIIIC), in structurally and functionally tuning Pol II transcription via chromatin looping and histone acetylation in human breast cancer cells [19]. TFIIIC recognizes the so-called A- and B-boxes of Pol III genes internal promoters [20]; these regulatory elements are also spread out in mammalian genomes within repetitive sequences (REs) such as Short INterspersed Elements (SINEs) Alu elements (AE) and Mammalian-wide Interspersed Repeats (MIRs) [21,22]. Many of these REs have cell-type specific expression [23,24] and participate in 3D genome folding [9,25,26]; therefore, it is tempting to speculate that TFIIIC might have the potential role to serve as a genome organizer in a cell type-specific fashion (like the CTFs) by virtue of its ability to bind these REs. However, the precise role of human TFIIIC in 3D genome shaping of different cell types remains unknown. Here, we use TFIIIC genome occupancy and Hi-C data from several human cell lines to explore the role of TFIIIC as a cell type-specific genome organizer. Our analysis shows that TFIIIC acts as a CTF by binding to cell type-expressed REs, which largely correspond to AE, but also to distinctive REs classes such as MIRs, FLAM-C and the cancer-enriched human alpha satellite (ALR/alpha), depending on the cell’s developmental origin. TFIIIC binding at these loci correlates with cell type-specific looping, which does not require colocalization with architectural proteins such as CTCF. Thus, our work defines a putative cell type-dependent architectural role for TFIIIC, and it can therefore be considered a as dual TF that serves as a CTF and a GTF in human cells.

## 2. Results

### 2.1. Human TFIIIC Shows Cell Type-Specific Genome Occupancy

To explore TFIIIC chromatin behavior in several human cell lines, we used TFIIIC genome-wide ChIP-seq data from fetal lung fibroblasts (IMR90), normal mammary gland epithelial cells (MCF10A), infiltrating ductal carcinoma breast cancer cells (T47D) [19], and human embryonic stem cells (hESCs) (see Materials and Methods for details). The presence of the TFIIIC complex on chromatin was detected with a rabbit polyclonal antibody raised against the N-terminal 477 amino acids of the second largest subunit of TFIIIC (TFIIIC110 or GTF3C2) [19].

**Figure 1:**
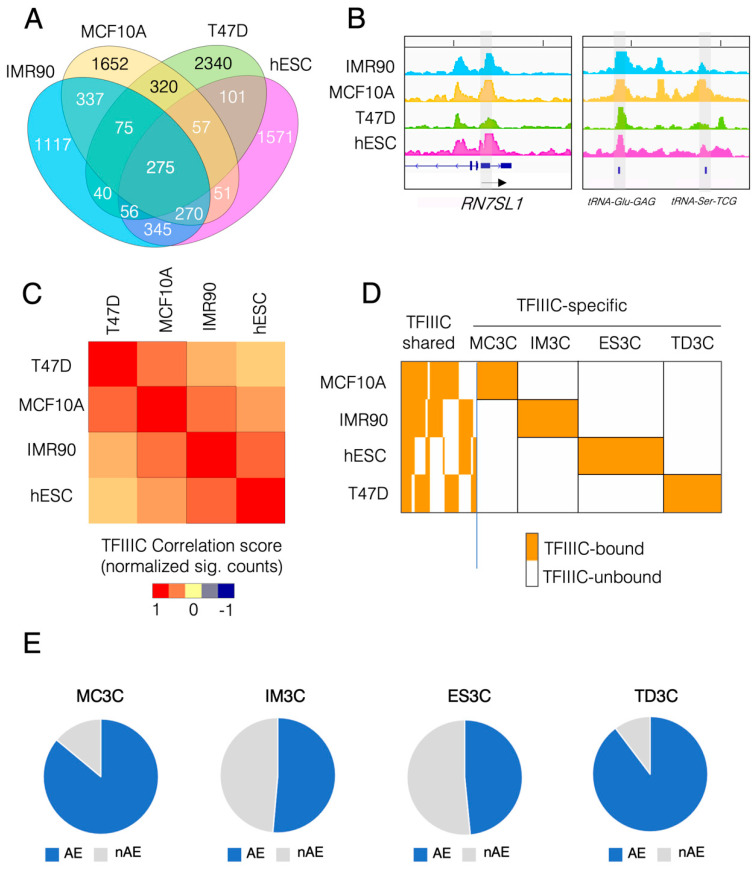
TFIIIC occupancy in several human cell lines displays marked cell type specificity and overlap with repetitive elements. (**A**) Venn diagram of the overlap among the genome-wide peaks for TFIIIC occupancy in the 4 human cell lines used in this study (IMR90, MCF10A, T47D and hESCs). (**B**) Genome browser view of ChIP-seq data for TFIIIC on two Pol III representative loci: the *RN7SL1* gene and the gene for the *tRNA-Glu-GAG* (arrow indicates the direction of transcription). Highlighted in grey is the gene bound by TFIIIC in all 4 cell lines. Note that the gene for the *tRNA-Ser-TCG* is only occupied in MCF10A. (**C**) Matrix of Pearson’s correlation between TFIIIC ChIP-seq data across the cell lines indicated. Color scale indicates the correlation score based on normalized sig tags. (**D**) Heatmap of combinatorial occupancy of ChIP-seq of TFIIIC across all cell lines indicated. Each vertical line represents a genomic coordinate which could be bound (orange color) or not bound (white color) by the factor. Left side of the figure shows the TFIIIC-shared sites across all the cell line tested. Cell type-specific occupancy of TFIIIC (right part of the heatmap) has been highlighted by the black boxes and each set of genomic coordinates particular for each cell line has been named accordingly: MCF10A (MC3C), IMR90 (IM3C), hESCs (ES3C) and T47D (TD3C). (**E**) Pie charts showing the fraction of MC3C, IM3C, ES3C and TD3C that overlap with AE (AE) in blue and not overlapping with AE (nAE) in grey.

First, we asked what was the degree of similarity among all the cell lines analyzed in terms of TFIIIC occupancy. To this end, we computed the peak’s overlap of all the significant TFIIIC peaks for all four cell lines and displayed the results as a Venn diagram (Figure 1A). Genome-wide, 275 sites were occupied by TFIIIC in all the cell lines analyzed (Figure 1A). Among these sites, we could find canonical Pol III genes such as *RNA7SL1* (the RNA of the signal recognition particle) and one unique tRNA gene (*tRNA-Glu-GAG*) (Figure 1B). Of these common sites, 218 mapped to AE, suggesting the existence of a core of AE capable of recruiting TFIIIC in a cell type-independent manner. We also calculated the genome-wide Pearson correlation for TFIIIC occupancy and displayed the results as a matrix heat map (Figure 1C). A larger degree of similarity was found between the breast samples (T47D and MCF10A) compared to the lung fibroblasts and hESCs and between normal cells (hESCs and IMR90) when compared to cancer cells (T47D) (Figure 1C). However, overall TFIIIC genome-wide occupancy showed that the vast majority of binding sites are cell type-specific: almost 50% for IMR90, 54% for MCF10A, 68% for T47D and 57% for hESCs (Figure 1A). In order to gain insight into these unique, cell type-specific TFIIIC binding sites, we calculated the multi-intersection of all TFIIIC maps using bedtools [27] and ranked the occupied regions (TFIIIC-bound vs. TFIIIC-unbound) in a combinatorial manner (Figure 1D). As in Figure 1A this analysis showed that at least 50% of all TFIIIC peaks were specific for each cell line. We named these cell line-specific TFIIIC-bound regions MC3C, IM3C, ES3C and TD3C for MCF10A, IMR90, hESCs and T47D cells, respectively (Figure 1D).

Next, we implemented ChIP-Enrich [28] to perform gene ontology (GO) analysis of each cell line specific TFIIIC-bound regions. The analysis highlighted the distinct genomic TFIIIC profile on Pol II genes for each cell line (Supplementary Figure S1). For instance, the MC3C regions showed significant (adjusted *p*-value < 0.001) enrichment for genes associated with cadherin adhesion (Supplementary Figure S1A), which are normally downregulated when MCF10A undergo epithelial-mesenchymal transition [29]. ES3C were specifically associated with terms related to stem cells such as beta-catenin/TCF-complex assembly (Supplementary Figure S1B) [30,31]. IM3C were associated with genes specific of fibroblast functions such as regulation of locomotion [32,33] (Supplementary Figure S1C). Finally, TD3C were found to be enriched in genes related to epithelial cell polarity [34] and response to hypoxia, as this pathway promotes tumor escaping and metastasis [35] (Supplementary Figure S1D). Indeed, T47D cells are derived from a metastatic pleural effusion from a patient with an infiltrating ductal carcinoma of the breast. All together these data show that human TFIIIC occupies loci associated with genes related to cell type-specific functions.

### 2.2. TFIIIC-Bound Regions Are Dominated by Alu Elements, but Also Contain Cell Line-Specific Repetitive Elements

**Figure 2:**
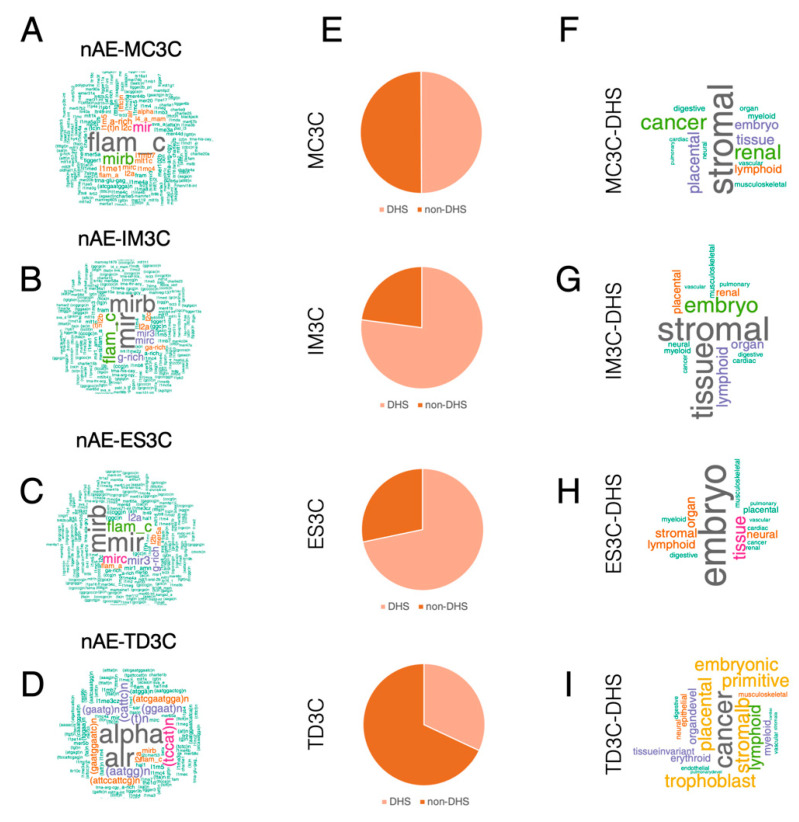
Cell line-specific TFIIIC-bound regions are DHSs linked with phenotypic traits. (**A**–**D**) Word Cloud analysis of REs associated with non-AE (nAE) MC3C, IM3C, ES3C. Larger fonts stand for higher frequency of the RE. (**E**) Pie charts showing the fraction of MC3C, IM3C, ES3C and TD3C that overlap with DHSs as defined in [36] in light orange and non-overlapping (dark orange). (**F**–**I**) Word Cloud analysis of disease and phenotypic traits of MC3C, IM3C, ES3C and TD3C that overlap with DHSs (MC3C-DHS, IM3C-DHS, ES3C-DHS and TD3C-DHS) as defined in [36]. Larger fonts stand for higher frequency of the disease and phenotypic traits found in the overlap.

As TFIIIC binds REs such as AE and MIR [20,23,24], we then asked whether TFIIIC-bound regions could display some cell-type specificity in terms of RE enrichment. Thus, we intersected TFIIIC-bound regions with all the REs of the human genome and found that each cell-specific dataset showed a marked overlap with AEs (Figure 1E). MC3C and TD3C showed the largest fraction of AE enrichment, whereas in ES3C and IM3C the AE-overlapping fraction represented around 50% of the loci (Figure 1E). We then asked whether there was a differential TFIIIC occupancy between AE and non-AE loci. The level of TFIIIC binding showed no differences between the datasets in any of the cell lines analyzed (Supplementary Figure S2A), attesting to a similar affinity of TFIIIC for regions of AE and regions of non-AE identity. We then wondered whether the non-AE regions show a specific enrichment for any particular RE. To answer this question, we compared all known REs of the human genome with each nAE set for overlap and extracted the name of the overlapping RE. The analysis showed that MC3C displayed a strong enrichment in FLAM-C elements (Figure 2A), whereas ES3C and IM3C non-AE showed a marked enrichment in MIR elements (Figure 2B,C). Lastly, the TD3C non-AE fraction was mostly constituted by ALR/Alpha elements (Figure 2D). Interestingly, hESCs and IMR90 are cells of non-epithelial origin, whereas MCF10A and T47D have indeed an epithelial origin. All together these data suggest that TFIIIC-bound regions largely overlap with AE, but also with other REs whose type appears to depend on the cell’s developmental origin.

### 2.3. Both AE and Non-AE TFIIIC-Bound Regions Are DHSs Associated with Cell Line-Specific Diseases and Phenotypic Traits

DNase I hypersensitive sites (DHSs) are cis genetic fingerprints of regulatory DNA elements shown to possess cell type- and state-specificity in humans [36]. In addition, DHSs contain genetic variations associated with phenotypic traits [36,37]. The recent creation of a comprehensive census of DHSs on a wide collection of human cells accurately associated with biological phenotypes [38] allowed us to ask whether the cell type-specific TFIIIC-bound regions could be linked to cell type identity. First, we overlapped the MC3C, IM3C, ES3C and TD3C datasets with the human DHSs and found that the majority of TFIIIC-bound regions overlapped with annotated DHSs, with the exception of TD3C, for which only 32% of the regions could be considered DHSs (Figure 2E). This differential behavior could be due to the transformed nature of the T47D cells compared with the other cell lines. Moreover, when we analyzed the phenotypes associated with the DHSs-overlapping regions, we found that both MC3C and IM3C were associated with “stroma” (Figure 2F,G), in agreement with fibroblasts being indeed the predominant cells in stroma [39]. IM3C were also enriched for “embryo” (Figure 2G), concurring with the fetal origin of IMR90 cells. ES3C were largely associated with embryonic DHSs (Figure 2H). Lastly, TD3C were majorly enriched in “cancer” phenotypes (Figure 2I). Based on these data, we conclude that cell line specific TFIIIC-bound regions MC3C, IM3C, ES3C and TD3C contain a large portion of DHSs linked to the establishment of cell line phenotypes.

As cell type-specific TFIIIC-bound regions are both AE and non-AE, we wondered which group contributed most to the overlap with DHSs. The intersected of the different datasets showed that the AE fraction provided the greatest contribution to the DHS overlap for IM3C and ES3C, with 809 DHS-positive regions out of 1037 AE (78%) and 985 DHS-positive regions out of 1162 AE (85%), respectively (Supplementary Figure S2B,C). The overlap for the non-AE was slightly lower (p-value fisher exact test = 0.0078), with 636 DHS-positive regions out of 981 non-AE (65%) for IM3C and 756 DHS-positive regions out of 1236 non-AE (61%) for ES3C (Supplementary Figure S2B,C). An inverse trend was observed for the MF3C and TD3C sites, with the non-AE regions largely contributing to the DHSs’ overlap (Supplementary Figure S2D,E). Indeed, the non-AE-MF3C regions DHS-positive were 288 out of 310 total (92%) and 53% for non-AE-TD3C (161 out of 304 regions) (Supplementary Figure S2D,E). The overlap was lower for the AE regions for both cell types: 1013 over a total of 1909 (57%) in MF3C and 951 out of 2650 (35%) in TD3C (Supplementary Figure S2D,E). Therefore, we conclude that AE-TFIIIC-bound regions largely correspond to DHSs in human primary fibroblasts and human embryonic stem cells (IM3C and ES3C), while non-AE have a higher percentage of overlap with DHSs for cells of breast epithelial origin such as MCF10A and T47D (MC3C and TD3C). Nonetheless, by looking at absolute numbers for all four cell lines combined, AE-TFIIIC-bound regions represent a total of 57% DHSs (3848 regions over a total of 6758 AE) whereas nAE-TFIIIC-bound regions constitute 65% of DHSs (1841 regions compared to a total of 2831). All these data point to the fact the both AE and non-AE regions are part of DHSs in the human genome and may therefore contribute to the acquisition of the phenotype for a particular cell type.

### 2.4. Long-Range Interactions of TFIIIC-Bound Regions Mediate Chromatin Looping in a Cell Type-Specific Manner

**Figure 3:**
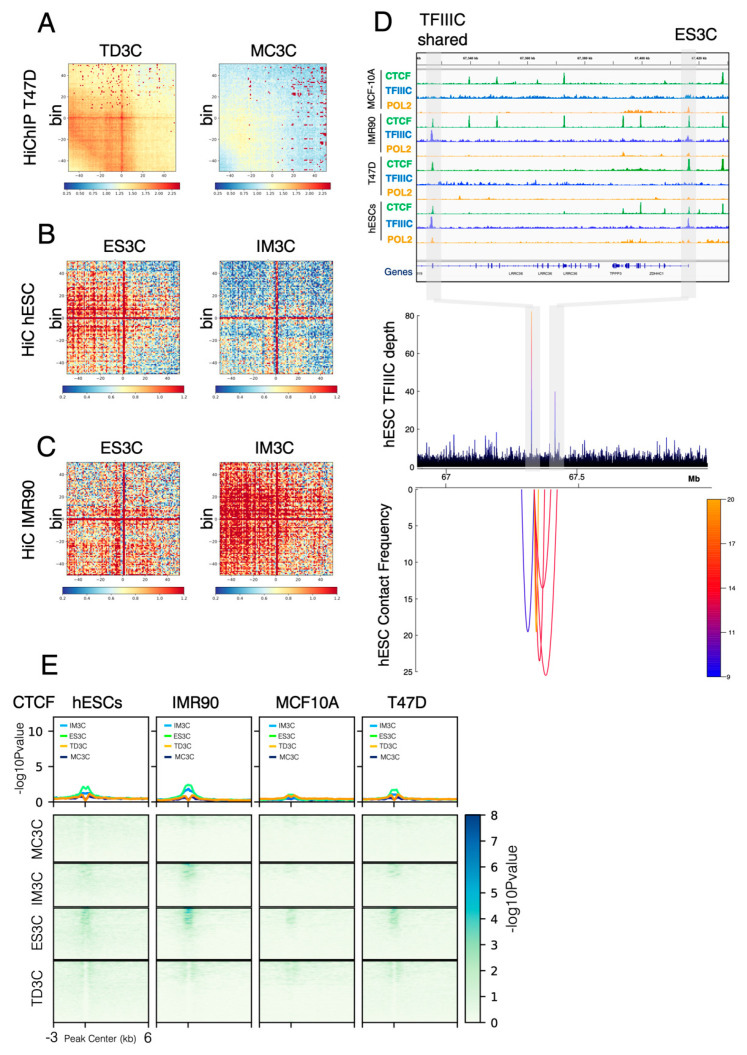
Long-range chromatin loops formed by TFIIIC-bound regions are cell type-specific and devoid of CTCF. (**A**) HiCExplorer Aggregate plots. Long distance interactions among TD3C and MF3C using T47D HiChIP data from our previous study [42]. Hi-C analysis showed increased contacts among TD3C than among MF3C. The genomic coordinates of the TD3C and MF3C are centered between half the number of bins and the other half number of bins. Plotted are the sub-matrices of the aggregated contacts frequency for 40 bins (1.5 kb bin size, 60 kb in total) both upstream and downstream direction. Color bar scale with increasing red shades of color stands for higher contact frequency. (**B**) HiCExplorer Aggregate plots. Long distance interactions in ES3C and IM3C using hESCs Hi-C data from [41]. Hi-C analysis showed increased contacts in ES3C as compared with in IM3C. (**C**) HiCExplorer Aggregate plots. Long distance interactions in ES3C and IM3C using IMR90 Hi-C data from [41]. Hi-C analysis showed increased contacts in IM3C as compared with in ES3C. (**D**) Interaction map for Hi-C data at the 100 kb LRRC36 locus in hESCs. Top panel: ChIP-seq data for the indicated factors (CTCF, Pol2 and TFIIIC) in each cell line used in this study. Bottom panel: interaction frequency map obtained by the loop caller FitHiChIP [43] on hESCs Hi-C data [41]. Arcs indicate the interaction between different genomic coordinates and colors reflect the intensity of the interaction. The location of the ES3C and TFIIIC shared sites is indicated by the grey rectangles. (**E**) Heatmap of -log10 of the Poisson *p*-value for CTCF occupancy in MC3C, IM3C, ES3C and TD3C for each cell line used in this study. Very low enrichment is detected for these cell type-specific regions of TFIIIC binding in all the cell lines tested. Color map is indicated.

We have previously reported that TFIIIC controls gene expression via mechanisms involving gene looping and histone acetylation in response to serum starvation in T47D cells [19]. As many DHSs are important cis-regulatory elements that can be part of enhancers or super-enhancers [5] and given that TFIIIC is a bona fide genome organizer [19,40], it is tempting to speculate that the TFIIIC-bound regions might be involved in mechanisms sustaining long range chromatin looping. To address this point, we used published Hi-C data sets IMR90 and hESCs [41] and Hi-C and HiChIP data in T47D [19,42]. To prove that TFIIIC-bound regions are involved in long-range chromatin contacts we therefore moved to identity loops in our Hi-C data and HiChIP data using loop caller FitChIIP [43]. First, we used TD3C regions to explore the degree of global contacts in T47D HiChIP data. Aggregation plots show that TD3C regions displayed higher contact density than MF3C regions (Figure 3A), although both sets of genomic TFIIIC coordinates are coming from cell lines of breast epithelial origins, indicating a large degree of cell specificity in chromatin looping made by TFIIIC. We then hypothesized that a similar behavior should be observed when comparing Hi-C data of IMR90 and hESCs to the corresponding IM3C and ES3C regions. Therefore, we compared aggregation plots from Hi-C data from hESCs for both sets of TFIIIC-bound regions and found that ES3C displayed stronger contact frequency compared to IM3C within Hi-C matrix generated with hESCs data (Figure 3B). We then took the same inverted approach and compared contact frequency of IM3C and ES3C regions in Hi-C data from IMR90 cells (Figure 3C). As expected IM3C showed the strongest degree of genome interactions compared to ES3C (Figure 3C). All together, these data prove how TFIIIC participates in the layout of chromatin looping in a cell type specific manner.

### 2.5. CTCF Is Not Required for Chromatin Looping at TFIIIC-Bound Regions

The most studied genome organizer is known as CCCTC-binding factor (CTCF) [19,40]. Therefore, we asked whether CTCF could be implicated in TFIIIC-bound cell type-specific chromatin looping. To measure this, we looked into T47D HiChIP data and found that TD3C sites are able to interact with each other forming long-range chromatin loops at the 1.5 Mb keratin locus of chromosome 17 (Supplementary Figure S3A). The HiChIP data show that two TD3C sites (not present in other cell lines) (Supplementary Figure S3A, upper panel) were more than 1.5 Mb apart but able to make chromatin contacts (Supplementary Figure S3A, lower panel), despite the absence of CTCF at these regions (Supplementary Figure S3A, upper panel), strongly suggesting a role for TFIIIC in promoting these contacts. Same results were obtained when we looked at another region of 100 kb containing the Leucine Rich Repeat Containing 36 (LRRC36) gene on chromosome 16 (Figure 3D). This loop was only present in hESCs and not in other cell lines (Figure 3D and Supplementary Figure S3B), independently of the presence of CTCF. Indeed, CTCF was present at both the ES3C and shared TFIIIC sites in hESCs, T47D and IMR90 cells (Figure 3D, upper panel). However, we were able to detect loops between the ES3C peak and the shared TFIIIC binding site only in hESCs where a specific ES3C appeared (Figure 3D and Supplementary Figure S3B), suggesting that this interaction largely depends on TFIIIC occupancy.

To further prove that CTCF is not involved in looping generated at cell type-specific TFIIIC-bound regions, we calculated the average profile of CTCF at MC3C, IM3C, ES3C and TD3C. We found that CTCF was indeed not significantly enriched at any of the cell type-specific TFIIIC-bound regions (Figure 3E). A slight enrichment was detected for IM3C and ES3C (Figure 3E), which harbor the largest fraction of DHS sites (Supplementary Figure S2A). On the contrary, CTCF enrichment was higher at TFIIIC-shared sites, which contain several known Pol III-transcribed genes and a vast majority of tRNA genes across cell lines (Supplementary Figure S3C). All together these data show that in normal conditions CTCF is not directly involved in chromatin loops associated with TFIIIC-bound regions that show cell type-specificity.

## 3. Discussion

3D genome organization plays a fundamental role in regulating gene transcription [44,45]. However, very little is known about the contribution of transcription factors and in particular whether GTFs together with CTFs can also participate in organizing the structural landscape of nuclear chromatin. Here, we provide a detailed analysis of the involvement of the Pol III GTF TFIIIC in the organization of chromatin structure via looping formation at DEEs in a cell type-specific manner. This study therefore supports the view that even a GTF, which is expressed in all cells within the human body, could actually provide a function related to 3D genome organization typical of master regulators of transcription such as CTFs. Our findings indicate that a large fraction of TFIIIC genomic occupancy is cell type-specific (at least for the cell lines used in the study), whereas shared loci concur with the TFIIIC general function in favoring transcription of its known Pol III gene targets and consistent across all cell lines examined. These results are in agreement with previous data reported by our group [19] and others [46]. The cell type-specific TFIIIC sites (MC3C, IM3C, ES3C and TD3C) are associated with GO terms in line with basic functions of the cell type they belong to, suggesting that these target sites might aim to determine and/or sustain cell-specialized cis-regulatory functions. Supporting this regulatory role, TFIIIC-bound regions are largely composed of REs and, in particular AE, which represent a large portion of transposable elements and have been adapted to evolve proto-enhancers function in the human genome [47]. On the same line, our previous work showed that AE bound by TFIIIC could provide a new way of regulating spatial nuclear localization of key cell cycle genes impacting their expression, and linked to clinical outcome in breast cancer patients [19]. In addition, each set of cell type-specific TFIIIC sites also harbors a particular group of non-AE, as the case of MIRs in hESCs and IMR90. Notably, these elements have been shown to possess a widespread role of enhancer-like transposable elements in cell identity and long-range genomic interactions [48]. In the case of MCF10A, the non-AE TFIIIC sites overlapped with FLAM-C elements, which are Alu monomers and shown to be a predictor of breast ductal carcinoma [49]. This could be compatible (and a hypothesis to test) with a putative role of these elements in providing new cis-regulatory elements to push immortalization of normal MCF10A breast cells. The small fraction of non-AE TFIIIC sites in T47D were constituted predominantly by ALR/Alpha, a cancer-enriched human alpha satellite, in line with a great expansion of these elements in tumor cells [50].

TFIIIC recognizes two DNA sequences known as A- and B-box, which are the prototypical internal promoter of Pol III transcriptional units [20], and are also contained within AE. In fact, we have shown that AEs serve as cis-regulatory landing platforms for the six-subunit TFIIIC factor [19]. Following local chromatin alterations, TFIIIC can create new loops (or reinforce pre-existing ones) to maintain cell cycle gene expression in T47D breast cancer cells [19]. In this work we extend this concept to three other cell lines. Our data provide evidence that cell line specific TFIIIC-bound regions highly overlap with DHSs, well-known markers of mostly distal enhancer regulatory elements (DEEs). DHSs are tied-up with the establishment of cell phenotype and shown to be enriched in both trait heritability and disease-associated variants [38,51]. Therefore, it is possible that cell type-specific TFIIIC sites could behave DEEs capable of triggering loop formation in distinct cellular contexts. Our data clearly point to this direction as shown by the ability of TFIIIC-bound regions to create chromatin loops only in the cell type they appear. This is a striking observation considering that this job was normally attributed to CTFs and/or certain 3D genome architectural proteins such as CTCF and cohesin [12,52]. Indeed, TFIIIC was previously suggested to contain functional similarities to both general transcription factors and transcriptional activators [53]. Our data also provide evidence that looping-forming TFIIIC-bound regions are devoid of CTCF sites and that TFIIIC sites shared across cell lines have higher level of occupancy for CTCF than at cell type-specific TFIIIC-bound regions. Notably, as we previously reported [19], other proteins might also be involved in GTF3C-mediated looping, such as the Activity dependent neuroprotective protein (ADNP) [19,54]. However, in the absence of ChIP-seq data for ADNP and other possible TFIIIC interactors, we cannot rule out a scenario where TFIIIC action at cell type-specific TFIIIC sites could be potentially sustained by other factors.

Finally, a general conclusion of our findings is that cis-regulatory elements within the mammalian genome are not such well-defined entities as previously thought, and many more TFs, which have been mis-categorized as housekeeping GTFs, such as TFIIIC, might instead play more cell-type specific functions in the DEEs and promoters’ interaction.

## 4. Materials and Methods

### 4.1. H9 Cell Culture

Human H9 (WiCell) ES cells were grown feeder-free on the matrigel (Corning™ 354277, ThermoFisher Scientific, Waltham, MA, USA) coated 6-well plate in the maintenance media (StemCell Technologie # 85850, Vancouver, Canada). They were passaged with enzyme-free method (ReLeSR, StemCell Technologie #05872, Vancouver, Canada) once they become nearly confluent. On the day of passaging, the media is supplemented with 10 µM Y27632 (Rho kinase inhibitor IV, EMD Chemicals, Merck KGaA, Darmstadt, Germany).

### 4.2. Chromatin Immunoprecipitation (ChIP): Chromatin Purification and Library Preparation

Cross-linked chromatin free of RNA from hESC H9 was prepared in the lab of Atsushi Nakano at the University of California Los Angeles (UCLA), Los Angeles (USA), and immunoprecipitation was carried out with the TFIIIC antibody [19] and performed as previously described [25]. DNA was quantified with the Qubit HS kit Q32851 (ThermoFisher Scientific, Waltham, MA, USA).

### 4.3. ChIP-Seq Sequencing

Single-end sequencing libraries were constructed from 1 ng of immunoprecipitated and input DNA using the Ovation Ultralow DR Multiplex System 1–8 and 9–16 kit (NuGen). To minimize false positives calling, several input libraries were sequenced to reach saturation with a coverage of 4 reads/bp of the human genome for each condition.

### 4.4. External Data Sources

ChIP-seq data for GTF3C in IMR90, MCF-10A and T47D was taken from GEO: GSE120162. ChIP-seq for GTF3C in hESCs was produced in this study (deposited at GEO repository with ID number: GSE195499). ChIP-seq for CTCF in T47D was taken from GEO: GSE120162; in MCF-10A from GEO: GSM2599084; in IMR90 from GEO: GSM935404; in hESCs from GEO: GSM803419. ChIP-seq for Pol2 in T47D was taken from GEO: GSM1503827; in MCF-10A from GEO: GSM2467771; in IMR90 from GEO: GSM935513; in hESCs from GEO: GSM3395077.

### 4.5. ChIP-Seq Data Analysis

Analysis of ChIP-seq data was carried out as previously described [26] with minor modifications [27]. Heatmaps and average profiles of ChIP-seq data were generated using computematrix and plotheatmap function of deeptools 3.0 [28].

### 4.6. Bedtools

Bed intersection was carried out using bedtools [29] “intersectBed” function with default parameters of 1-bp overlap. Graphic representation of Venn diagrams has been obtained with R graphic, using R-studio (https://www.rstudio.com, accessed on 22 January 2022).

### 4.7. GO Analysis

ChIP-seq Enrich (http://chip-enrich.med.umich.edu, accessed on 22 January 2022) [30] was used with default parameters to detect GO terms enrichments and association of peaks with genes, respectively. Lists of genome coordinates derived from downstream analysis of ChIP-seq data were analyzed with ChIP-seq Enrich. Parameters used: locus definition (Nearest TSS), Enrichment Method (Chip-Enrich), filter (2000 genes), adjust for the mappability of the gene locus regions (False).

### 4.8. HiChIP-Seq Analysis

HiChIP-seq analysis was performed using dovetail HiChIP pipeline using manufacturer’s instructions (https://hichip.readthedocs.io/en/latest/index.html, accessed on 26 January 2022). Hi-C matrixes were generated from HiChIP-seq data using HiC-pro pipeline [31]. Finally, loops were called using the all to all parameters of FitHiChIP [32]. Loops representation was done using Sushi R script (https://bioconductor.org/packages/release/bioc/html/Sushi.html, accessed on 22 January 2022) and aligned with ChIP-seq data and Hi-C matrices. Finally, hicAggregateContacts plots were generated using HiCExplorer tools [33].

## 5. Conclusions

This work extends previous knowledge about the role of TFIIIC as a bona fide genome organizer whose action participates in cell type-dependent 3D genome looping via binding to REs. This work also emphasizes the role of diverse REs in regulating chromatin folding defying an architectural role for SINEs, FLAM-C, MIR, ALR/alpha in human cells via binding of TFIIIC. Our findings expose novel roles for this general transcription factor that could go far beyond its known function in Pol III transcription. Therefore, our data points to TFIIIC as a potential new “integrator” capable of reading different and dispersed REs within the genome and rewiring them into a cell type-specific network of CTCF-devoted loops in human cells.

## Data Availability

Sequencing data of TFIIIC ChIP-seq in H9 hESCs has been deposited in GEO: GSE195499.

## Supplementary Materials

The following supporting information can be downloaded at: https://www.mdpi.com/article/10.3390/ijms23042260/s1.

## References

[1] Zheng H., Xie W. (2019). The role of 3D genome organization in development and cell differentiation. Nat. Rev. Mol. Cell Biol..

[2] Li Y., Hu M., Shen Y. (2018). Gene regulation in the 3D genome. Hum. Mol. Genet..

[3] Lambert S.A., Jolma A., Campitelli L.F., Das P.K., Yin Y., Albu M., Chen X., Taipale J., Hughes T.R., Weirauch M.T. (2018). The human transcription factors. Cell.

[4] Juven-Gershon T., Kadonaga J.T. (2010). Regulation of gene expression via the core promoter and the basal transcriptional machinery. Dev. Biol..

[5] Schoenfelder S., Fraser P. (2019). Long-range enhancer-promoter contacts in gene expression control. Nat. Rev. Genet..

[6] Grosveld F., van Staalduinen J., Stadhouders R. (2021). Transcriptional regulation by (super) enhancers: From discovery to mechanisms. Annu. Rev. Genom. Hum. Genet..

[7] Stadhouders R., Vidal E., Serra F., Di Stefano B., Le Dily F., Quilez J., Gomez A., Collombet S., Berenguer C., Cuartero Y. (2018). Transcription factors orchestrate dynamic interplay between genome topology and gene regulation during cell reprogramming. Nat. Genet..

[8] Reese J.C. (2003). Basal transcription factors. Curr. Opin. Genet. Dev..

[9] Pombo A., Dillon N. (2015). Three-dimensional genome architecture: Players and mechanisms. Nat. Rev. Mol. Cell. Biol..

[10] Ren B., Yue F. (2015). Transcriptional enhancers: Bridging the genome and phenome. Cold Spring Harbor Symposia on Quantitative Biology.

[11] Merkenschlager M., Nora E.P. (2016). CTCF and cohesin in genome folding and transcriptional gene regulation. Annu. Rev. Genom. Hum. Genet..

[12] Weintraub A.S., Li C.H., Zamudio A.V., Sigova A.A., Hannett N.M., Day D.S., Abraham B.J., Cohen M.A., Nabet B., Buckley D.L. (2017). YY1 is a structural regulator of enhancer-promoter loops. Cell.

[13] Soutourina J. (2018). Transcription regulation by the Mediator complex. Nat. Rev. Mol. Cell Biol..

[14] Tippens N.D., Vihervaara A., Lis J.T. (2018). Enhancer transcription: What, where, when, and why?. Genes Dev..

[15] Andersson R., Sandelin A., Danko C.G. (2015). A unified architecture of transcriptional regulatory elements. Trends Genet..

[16] Ji X., Dadon D.B., Abraham B.J., Lee T.I., Jaenisch R., Bradner J.E., Young R.A. (2015). Chromatin proteomic profiling reveals novel proteins associated with histone-marked genomic regions. Proc. Natl. Acad. Sci. USA.

[17] Engelen E., Brandsma J.H., Moen M.J., Signorile L., Dekkers D.H., Demmers J., Kockx C.E., Ozgür Z., Van Ijcken W.F., Van Den Berg D.L. (2015). Proteins that bind regulatory regions identified by histone modification chromatin immunoprecipitations and mass spectrometry. Nat. Commun..

[18] Mizzen C.A., Yang X.J., Kokubo T., Brownell J.E., Bannister A.J., Owen-Hughes T., Workman J., Wang L., Berger S.L., Kouzarides T. (1996). The TAF(II)250 subunit of TFIID has histone acetyltransferase activity. Cell.

[19] Ferrari R., de Llobet Cucalon L.I., Di Vona C., Le Dilly F., Vidal E., Lioutas A., Oliete J.Q., Jochem L., Cutts E., Dieci G. (2020). TFIIIC binding to alu elements controls gene expression via chromatin looping and histone acetylation. Mol. Cell..

[20] Dieci G., Fiorino G., Castelnuovo M., Teichmann M., Pagano A. (2007). The expanding RNA polymerase III transcriptome. Trends Genet..

[21] Deininger P. (2011). Alu elements: Know the SINEs. Genome Biol..

[22] Ferrari R., Grandi N., Tramontano E., Dieci G. (2021). Retrotransposons as drivers of mammalian brain evolution. Life.

[23] Carnevali D., Dieci G. (2017). Identification of RNA polymerase III-transcribed SINEs at single-locus resolution from RNA sequencing data. Non-Coding RNA.

[24] Conti A., Carnevali D., Bollati V., Fustinoni S., Pellegrini M., Dieci G. (2015). Identification of RNA polymerase III-transcribed Alu loci by computational screening of RNA-Seq data. Nucleic Acids Res..

[25] Cournac A., Koszul R., Mozziconacci J. (2016). The 3D folding of metazoan genomes correlates with the association of similar repetitive elements. Nucleic Acids Res..

[26] Van De Werken H.J., Haan J.C., Feodorova Y., Bijos D., Weuts A., Theunis K., Holwerda S.J., Meuleman W., Pagie L., Thanisch K. (2017). Small chromosomal regions position themselves autonomously according to their chromatin class. Genome Res..

[27] Quinlan A.R., Hall I.M. (2010). BED tools: A flexible suite of utilities for comparing genomic features. Bioinformatics.

[28] Welch R.P., Lee C., Imbriano P.M., Patil S., Weymouth T.E., Smith R.A., Scott L.J., Sartor M.A. (2014). ChIP-Enrich: Gene set enrichment testing for ChIP-seq data. Nucleic Acids Res..

[29] Yuki K., Yoshida Y., Inagaki R., Hiai H., Noda M. (2014). E-cadherin-downregulation and RECK-upregulation are coupled in the non-malignant epithelial cell line MCF10A but not in multiple carcinoma-derived cell lines. Sci. Rep..

[30] Lechler T. (2012). Adherens junctions and stem cells. Adherens Junctions Mol. Mech. Tissue Dev. Dis..

[31] Yi F., Merrill B.J. (2007). Stem cells and TCF proteins: A role for beta-catenin--independent functions. Stem Cell Rev..

[32] Abercrombie M., Heaysman J.E., Pegrum S.M. (1970). The locomotion of fibroblasts in culture. 3. Movements of particles on the dorsal surface of the leading lamella. Exp. Cell Res..

[33] Bainbridge P. (2013). Wound healing and the role of fibroblasts. J. Wound Care.

[34] Van Deurs B., Zou Z.Z., Briand P., Balslev Y., Petersen O.W. (1987). Epithelial membrane polarity: A stable, differentiated feature of an established human breast carcinoma cell line MCF-7. J. Histochem. Cytochem..

[35] Hoffmann C., Mao X., Brown-Clay J., Moreau F., Al Absi A., Wurzer H., Sousa B., Schmitt F., Berchem G., Janji B. (2018). Hypoxia promotes breast cancer cell invasion through HIF-1alpha-mediated up-regulation of the invadopodial actin bundling protein CSRP2. Sci. Rep..

[36] Meuleman W., Muratov A., Rynes E., Halow J., Lee K., Bates D., Diegel M., Dunn D., Neri F., Teodosiadis A. (2020). Index and biological spectrum of human DNase I hypersensitive sites. Nature.

[37] Stergachis A.B., Neph S., Reynolds A., Humbert R., Miller B., Paige S.L., Vernot B., Cheng J.B., Thurman R.E., Sandstrom R. (2013). Developmental fate and cellular maturity encoded in human regulatory DNA landscapes. Cell.

[38] Maurano M.T., Humbert R., Rynes E., Thurman R.E., Haugen E., Wang H., Reynolds A.P., Sandstrom R., Qu H., Brody J. (2012). Systematic localization of common disease-associated variation in regulatory DNA. Science.

[39] Chen X., Song E. (2019). Turning foes to friends: Targeting cancer-associated fibroblasts. Nat. Rev. Drug Discov..

[40] Galli G.G., Carrara M., Francavilla C., Honnens de Lichtenberg K., Olsen J.V., Calogero R.A., Lund A.H. (2013). Genomic and proteomic analyses of Prdm5 reveal interactions with insulator binding proteins in embryonic stem cells. Mol. Cell. Biol..

[41] Dixon J.R., Selvaraj S., Yue F., Kim A., Li Y., Shen Y., Hu M., Liu J.S., Ren B. (2012). Topological domains in mammalian genomes identified by analysis of chromatin interactions. Nature.

[42] Zaurin R., Ferrari R., Nacht A.S., Carbonell J., Le Dily F., Font-Mateu J., de Llobet Cucalon L.I., Vidal E., Lioutas A., Beato M. (2021). A set of accessible enhancers enables the initial response of breast cancer cells to physiological progestin concentrations. Nucleic Acids Res..

[43] Bhattacharyya S., Chandra V., Vijayanand P., Ay F. (2019). Identification of significant chromatin contacts from HiChIP data by FitHiChIP. Nat. Commun..

[44] Schneider R., Grosschedl R. (2007). Dynamics and interplay of nuclear architecture, genome organization, and gene expression. Genes Dev..

[45] Le Dily F., Bau D., Pohl A., Vicent G.P., Serra F., Soronellas D., Castellano G., Wright R.H., Ballare C., Filion G. (2014). Distinct structural transitions of chromatin topological domains correlate with coordinated hormone-induced gene regulation. Genes Dev..

[46] Moqtaderi Z., Wang J., Raha D., White R.J., Snyder M., Weng Z., Struhl K. (2010). Genomic binding profiles of functionally distinct RNA polymerase III transcription complexes in human cells. Nat. Struct. Mol. Biol..

[47] Su M., Han D., Boyd-Kirkup J., Yu X., Han J.D. (2014). Evolution of Alu elements toward enhancers. Cell Rep..

[48] Cao Y., Chen G., Wu G., Zhang X., McDermott J., Chen X., Xu C., Jiang Q., Chen Z., Zeng Y. (2019). Widespread roles of enhancer-like transposable elements in cell identity and long-range genomic interactions. Genome Res..

[49] Beck J., Urnovitz H.B., Mitchell W.M., Schutz E. (2010). Next generation sequencing of serum circulating nucleic acids from patients with invasive ductal breast cancer reveals differences to healthy and nonmalignant controls. Mol. Cancer Res..

[50] Bersani F., Lee E., Kharchenko P.V., Xu A.W., Liu M., Xega K., MacKenzie O.C., Brannigan B.W., Wittner B.S., Jung H. (2015). Pericentromeric satellite repeat expansions through RNA-derived DNA intermediates in cancer. Proc. Natl. Acad. Sci. USA.

[51] Chen Y., Chen A. (2019). Unveiling the gene regulatory landscape in diseases through the identification of DNase I-hypersensitive sites. Biomed. Rep..

[52] Hansen A.S., Pustova I., Cattoglio C., Tjian R., Darzacq X. (2017). CTCF and cohesin regulate chromatin loop stability with distinct dynamics. Elife.

[53] Lata E., Choquet K., Sagliocco F., Brais B., Bernard G., Teichmann M. (2021). RNA polymerase III subunit mutations in genetic diseases. Front. Mol. Biosci..

[54] Ostapcuk V., Mohn F., Carl S.H., Basters A., Hess D., Iesmantavicius V., Lampersberger L., Flemr M., Pandey A., Thomä N.H. (2018). Activity-dependent neuroprotective protein recruits HP1 and CHD4 to control lineage-specifying genes. Nature.

