# Supplementary material for: An RNA Polymerase III General Transcription Factor Engages in Cell Type-Specific Chromatin Looping
Source: Int J Mol Sci. 2022 Feb 18;23(4):2260. doi: 10.3390/ijms23042260 (PMC8878802; doi:10.3390/ijms23042260)
Supplement: Supplementary file 1 [file ijms-23-02260-s001.zip › ijms-1593426-supplementary.pdf]

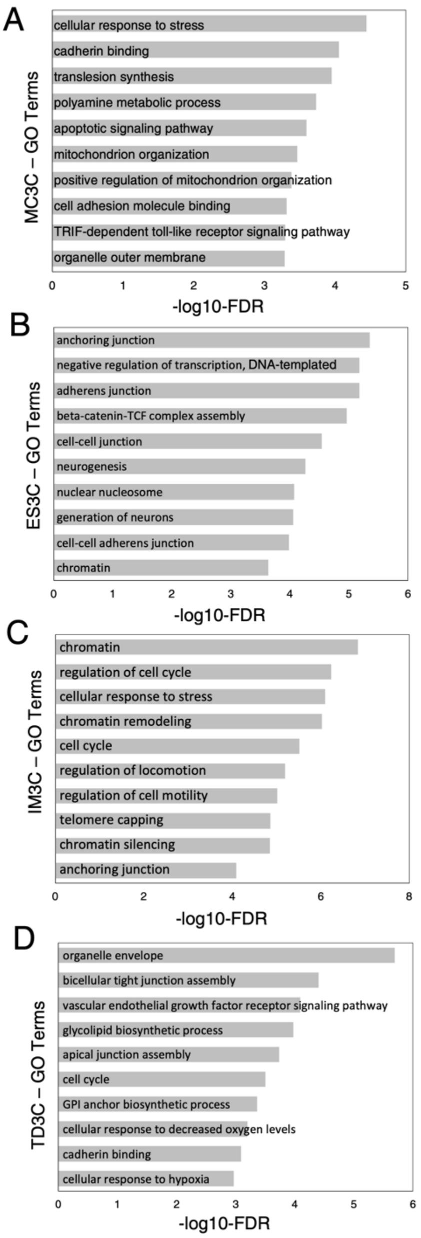

Supplementary Figure S1. GO enrichment of cell type-specific TFIIIC regions. A-D) ChIP-Enrich GO analysis of MC3C, IM3C, ES3C and TD3C regions. The bar graphs include the top ten enriched Biological Processes, Cellular Components and Molecular Functions terms with FDR<10^{-3} and ordered according to -log10(FDR).

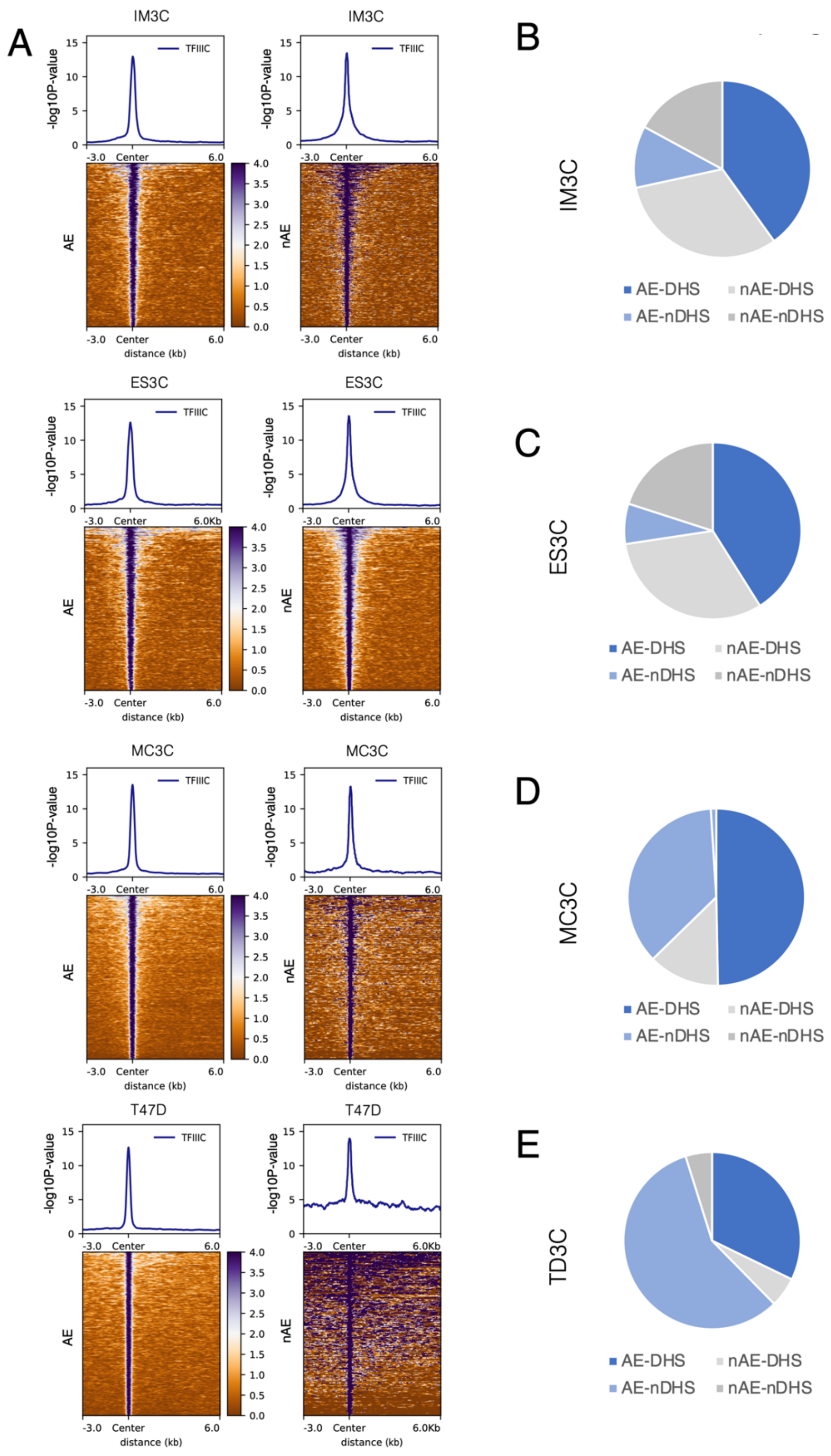

Supplementary Figure S2. AE and non-AE cell type specific TFIIIC-bound regions have differential overlap with DHS regions depending on the cell's developmental origin. A) Heatmap of -log10 of the Poisson *p*-value for TFIIIC occupancy over AE- and non-AE of the MC3C, IM3C, ES3C and TD3C datasets. A high and equal enrichment is detected for all cell

type-specific regions for TFIIIC binding in all the cell lines analyzed at both AE and non-AE loci. Color map is indicated. **B-E)** Pie charts showing the fraction of MC3C, IM3C, ES3C and TD3C that overlap with DHSs and AE (AE) and non-AE (nAE) or do not overlap with DHSs (nDHS) but overlap with AE and non-AE. Blue colors represent AE whereas grey colors represent non-AEs. The comparison shows that the percentage of AE and non-AE overlapping with DHS is similar for IMR90 and hESCs cells but different from MCF-10A and T47D, which are both lines of breast epithelial origin.

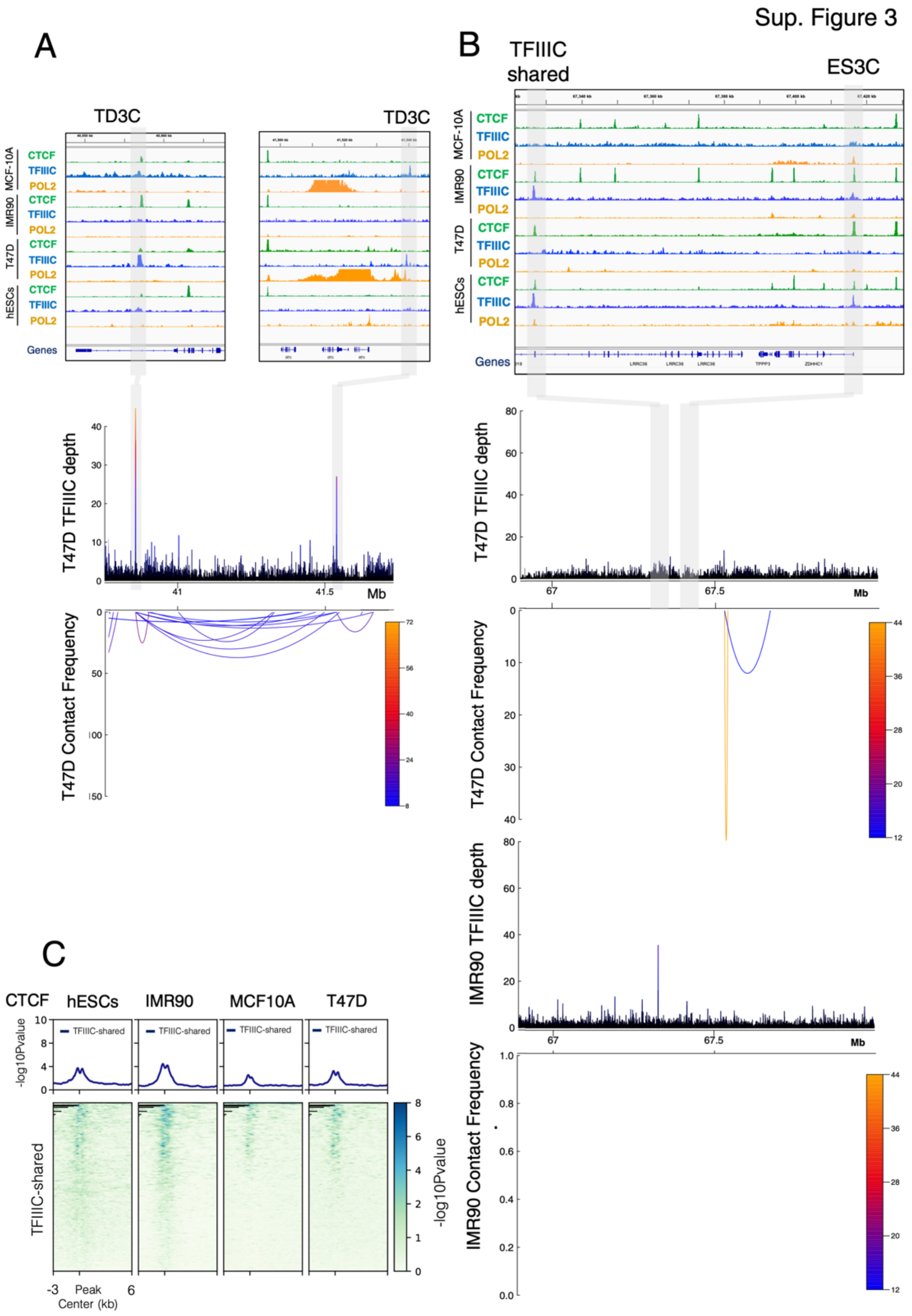

Supplementary Figure S3. Cell type-specific TFIIIC-bound regions form long range chromatin loops even in the absence of CTCF. A) Interaction map for Hi-C data at the 1.5 MB keratins locus in T47D cells. Top panel: ChIP-seq data for the indicated factors (CTCF, Pol2 and TFIIIC) in each cell line used in this study. Bottom panel: interaction frequency map obtained by the loop caller FitHiChIP [43] on T47D Hi-C data [19]. Arcs indicate the interaction

between different genomic coordinates and colors reflect the intensity of the interaction. The location of the two far apart TD3C sites is indicated by the grey rectangles. **B)** Interaction map for Hi-C data at the 100 kb *LRRC36* locus in hESCs as shown in Fig. 3D. Top panel: ChIP-seq data for the indicated factors (CTCF, Pol2 and TFIIIC) in each cell line used in this study. Bottom panel: interaction frequency map obtained by the loop caller FitHiChIP [43] on hESCs Hi-C data [41]. Below panel ChIP-seq depth tracks for TFIIIC in T47D and IMR90 are reported and two sets of arcs indicate the interaction between different genomic coordinates in the two cell lines. Arcs colors reflect the intensity of the interaction. For both T47D and IMR90 cells the absence of at least one TFIIIC site found in hESCs for the same region completely abrogates the chromatin looping detected in Fig. 3D. The location of the ES3C and TFIIIC shared sites is indicated by the grey rectangles. **C)** Heatmap of -log10 of the Poisson *p*-value for CTCF occupancy over TFIIIC-positive shared regions across all cell lines used in this study. Higher enrichment is detected for the shared TFIIIC-bound regions than for the cell type-specific TFIIIC sites (compared to Fig. 3E) in all the cell lines analyzed. Color map is indicated.
